# PD-L1 expression in angiomatoid fibrous histiocytoma

**DOI:** 10.1038/s41598-021-81746-y

**Published:** 2021-01-26

**Authors:** Joshua Byers, Hong Yin, Heather Rytting, Suzanna Logan, Mai He, Zhongxin Yu, Dehua Wang, Mikako Warren, Shamlal Mangray, Louis P. Dehner, Shengmei Zhou

**Affiliations:** 1 Department of Pathology and Laboratory Medicine, Children’s Hospital Los Angeles, MS 43, 4650 Sunset Boulevard, Los Angeles, CA 90027 USA; 2 Children’s Healthcare of Atlanta, Atlanta, GA USA; 3 Washington University in Saint Louis, Saint Louis, MO USA; 4 University of Oklahoma Health Sciences Center, Oklahoma City, OK USA; 5 Cincinnati Children’s Hospital, Cincinnati, OH USA; 6 Rhode Island Hospital, Providence, RI USA

**Keywords:** Diseases, Medical research

## Abstract

Angiomatoid fibrous histiocytoma (AFH) is a rare tumor of intermediate malignancy. Treatment options for unresectable and/or metastatic tumors are very limited. Immunotherapy with PD-1/PD-L1 inhibitors may be worth exploring. The aim of this study was to evaluate the expression of PD-L1 in AFHs. PD-L1 expression was assessed on 36 AFHs from 36 pediatric patients by immunohistochemical staining of PD-L1 (clone 22C3). Positivity was defined as membranous expression in ≥ 1% of either tumor or immune cells. The correlations between PD-L1 expression and clinicopathologic features were assessed. Two patients had lymph node metastasis. All patients underwent surgical resection; three of them also had systemic chemotherapy. Three patients had recurrence after initial resection; all patients were alive with a median follow-up of 2.5 years. Overall, twenty-two (61%) tumors were positively stained for PD-L1 and positivity was seen on both tumor and immune cells in eighteen of the 22 tumors. A positive correlation was found between tumor cell PD-L1 expression and CD8+ T-cell infiltration. There were no statistically significant differences between the status of PD-L1 expression and the clinicopathological features assessed. PD-L1 expression was identified in 61% of AFHs with a predominantly adaptive pattern. Our findings provide a rationale for future studies evaluating the potential of checkpoint immunotherapy for patients with unresectable and/or metastatic tumor.

## Introduction

Angiomatoid fibrous histiocytoma (AFH) is a rare “fibrohistiocytic” tumor of intermediate malignancy, mainly seen in children and young adults^1^. The most frequent site of occurrence is superficial soft tissues of the extremities, followed by trunk, and head and neck^2^. A variety of atypical sites have now been documented including brain, lung, mediastinum, retroperitoneum and adrenal gland^3–9^. The most common genetic aberration is an *EWSR1-CREB1* fusion, identified in > 90% of AFHs^10^. The majority of AFHs behave indolently, and complete surgical excision is the standard treatment; however local recurrence is noted in up to 15% of cases^11^. Albeit less frequently, a small subset of AFHs (less than 5%) metastasize, and distant metastasis is associated with mortality^1,12–15^. Treatment options for unresectable and or metastatic tumors are very limited^16,17^.

Notably, about 80% of AFHs are surrounded by a prominent lymphoid cuff, and most AFHs have dense lymphoplasmacytic infiltrates^2,18^. Furthermore, some AFH patients present with a paraneoplastic syndrome including fever, malaise, and anemia^1,12^. These features suggest that AFH is an immunogenic tumor, and immunotherapy targeting PD-1/PD-L1 may be useful for a subset of patients. PD-L1 (also known as B7-H1) is an important immune regulatory molecule; the binding of PD-L1 to its receptor PD-1 helps tumor cells evade anti-tumor immunity^19^. PD-L1 expression by tumor cells may represent a novel adaptive resistance mechanism of immune escape^20^. PD-L1 inhibitors have showed promising anti-tumor results in multiple clinical trials^21^. PD-L1 immunohistochemistry (IHC) has been utilized to identify those patients who are most likely to benefit from PD-1/PD-L1 inhibitors^22^. However, it remains unknown whether PD-L1 is expressed in AFH.

## Results

### Clinicopathologic characteristics

The clinicopathological features of each individual case are presented in Table 1. There were 21 females and 15 males with an age range of 2–15.5 years (median: 8 years). The primary tumor locations included upper extremities (12, 33%), head and neck (9, 25%), lower extremities (7, 19%), trunk (7, 19%) and adrenal gland (1, 3%). The tumor size ranged from 0.4 to 10.5 cm in greatest dimension.

**Table 1:** The clinicopathologic features of 36 cases.

Case	Sex	Age (yrs)	Tumor site	Max. tumor size (cm)	Molecular study	CD68	CD99	Desmin	Tumor cell PD-L1	Immune cell PD-L1	CD8+ T cells	Follow-up	Virtual status
1	F	14	UE	5.5	NA	+	NA	+	0	0	0	1mo	WD
2	M	7	UE	1	NA	NA	NA	NA	0	0	0	5 yr	NED
3	F	8	HN	2.5	NA	NA	NA	NA	0	0	0	1mo	NED
4	F	8	HN	2.6	NA	NA	NA	+	0	0	0	NA	NA
5	M	10	HN	5.1	NA	+	NA	0	0	0	3 +	NA	NA
6	M	12	LE	5.3	NA	+	+	+	1 +	1 +	0	NA	NA
7	F	11	LE	6.5	NA	+	NA	+	1 +	1 +	1 +	4.5 yr	NED
8	F	6	T	2.3	NA	+	+	NA	2 +	1 +	3 +	10 mo	NED
9	M	7	LE	1.3	NA	+	+	+	1 +	1 +	0	4.5 yr	NED
10	F	5	LE	1.2	NA	+	+	NA	1 +	1 +	0	2 yr	NED
11	M	11	UE	2.5	NA	0	+	NA	2 +	1 +	1 +	1 mo	NED
12	F	12	LE	1.1	*EWSR1* +	NA	NA	+	1 +	1 +	1 +	1.5 yr	NED
13	M	2	LE	2	*EWSR1* +	NA	NA	NA	3 +	1 +	2 +	4 mo	NED
14	F	9	T	4.5	*EWSR1*− and *FUS*−	+	+	+	0	1 +	0	4 mo	NED
15	F	10	HN	2.3	*EWSR1* +	NA	+	+	1 +	0	2 +	4 mo	NED
16	F	7	T	1.2	*EWSR1* +	0	NA	0	1 +	0	1 +	1.5 yr	NED
17	M	8	HN	1.6	*EWSR1*−*CREB1*	+	+	+	1 +	0	1 +	1 mo	NED
18	M	5	UE	2.3	*EWSR1*−*CREB1*	+	+	+	0	0	0	1 mo	NED
19	F	15.5	T	2	NA	+	NA	NA	0	0	0	1 mo	NED
20	F	7.5	HN	4.5	*EWSR1* +	+	NA	+	1 +	1 +	1 +	5 yr	NED
21	F	4	UE	2.5	NA	NA	NA	NA	0	0	0	5 yr	NED
22	M	13	UE	5	NA	+	NA	+	0	0	0	13 yr	NED
23	F	11	HN	1.2	*EWSR1* +	+	+	+	0	0	0	4 mo	NED
24	M	9	UE	1.6	NA	NA	NA	NA	0	0	0	4 mo	NED
25	F	14	Adrenal	10.5	*EWSR1-ATF1*	+	+	0	3 +	1 +	3 +	4 mo	WD
26	M	5	T	2.5	*EWSR1-CREB1*	+	+	0	0	0	0	5 yr	NED
27	F	8	T	1.2	*EWSR1-CREB1*	NA	+	+	3 +	1 +	1 +	5 mo	NED
28	F	5	T	2	NA	0	NA	+	1 +	1 +	1 +	11 yr	NED
29	M	8	UE	2.5	*EWSR1* +	+	NA	0	1 +	1 +	1 +	9 yr	NED
30	M	4	UE	1.8	*EWSR1* +	NA	NA	+	1 +	1 +	1 +	3 yr	NED
31	M	9	UE	4	*EWSR1* +	NA	NA	NA	1 +	1 +	1 +	2 yr	NED
32	F	12	HN	0.4	*EWSR1* +	+	+	NA	0	0	0	1 yr	NED
33	M	11	HN	3.5	NA	NA	NA	NA	1 +	1 +	1 +	4 yr	NED
34	F	6	UE	0.7	NA	+	+	NA	1 +	1 +	1 +	5 yr	NED
35	F	10	LE	1.1	NA	+	+	NA	0	0	0	5 mo	NED
36	F	8.5	T	1.2	*EWSR1-CREB1*	+	+	NA	3 +	1 +	3 +	1.5 yr	NED

F, female; M, male; UE, upper extremity; HN, head and neck; LE, lower extremity; T, trunk; NA, not available ; + , positive; 1 + , positive staining in 1% to 10% cells for PD-L1, 10–49 positive cells per 200 × field for CD8; 2 + , positive staining in 11% to 30% cells for PD-L1, 50–100 positive cells per 200 × field for CD8; 3 + , positive staining in > 30% cells for PD-L1, > 100 positive cells per 200 × field for CD8; mo, month; yr, year; WD, with disease; NED, no evidence of disease.

Positive *EWSR1* rearrangement was seen in 12/13 tested by FISH including two cases with an *EWSR1-CREB1* fusion. One case was negative for both *EWSR1* and *FUS* by FISH. By OncoKids^SM^, three cases showed an *EWSR1-CREB1* fusion and one case with an *EWSR1-ATF1* fusion. No other clinically significant gene mutation or gene amplification events were identified in these four cases. CD99 positivity was seen in 17/17 cases tested. Most cases were also positive for CD68 (23/24) and desmin (16/21).

Four patients had a paraneoplastic syndrome. All tumors underwent surgical resection. Three patients had recurrence after initial resection, and two of three patients underwent re-excision with no evidence of residual disease at last follow-up. Two patients had lymph node metastasis at the time of diagnosis. Three patients also received chemotherapy. All patients were alive with a median follow-up of 2.5 years (range: 1 month to 13 years). Two patients were alive with disease at the last follow-up. Twenty-four (65%) tumors showed typical peripheral lymphoid cuffing and all tumors demonstrated intratumoral lymphoplasmacytic infiltrates.

### PD-L1 expression on tumor and immune cells

**Figure 1:**
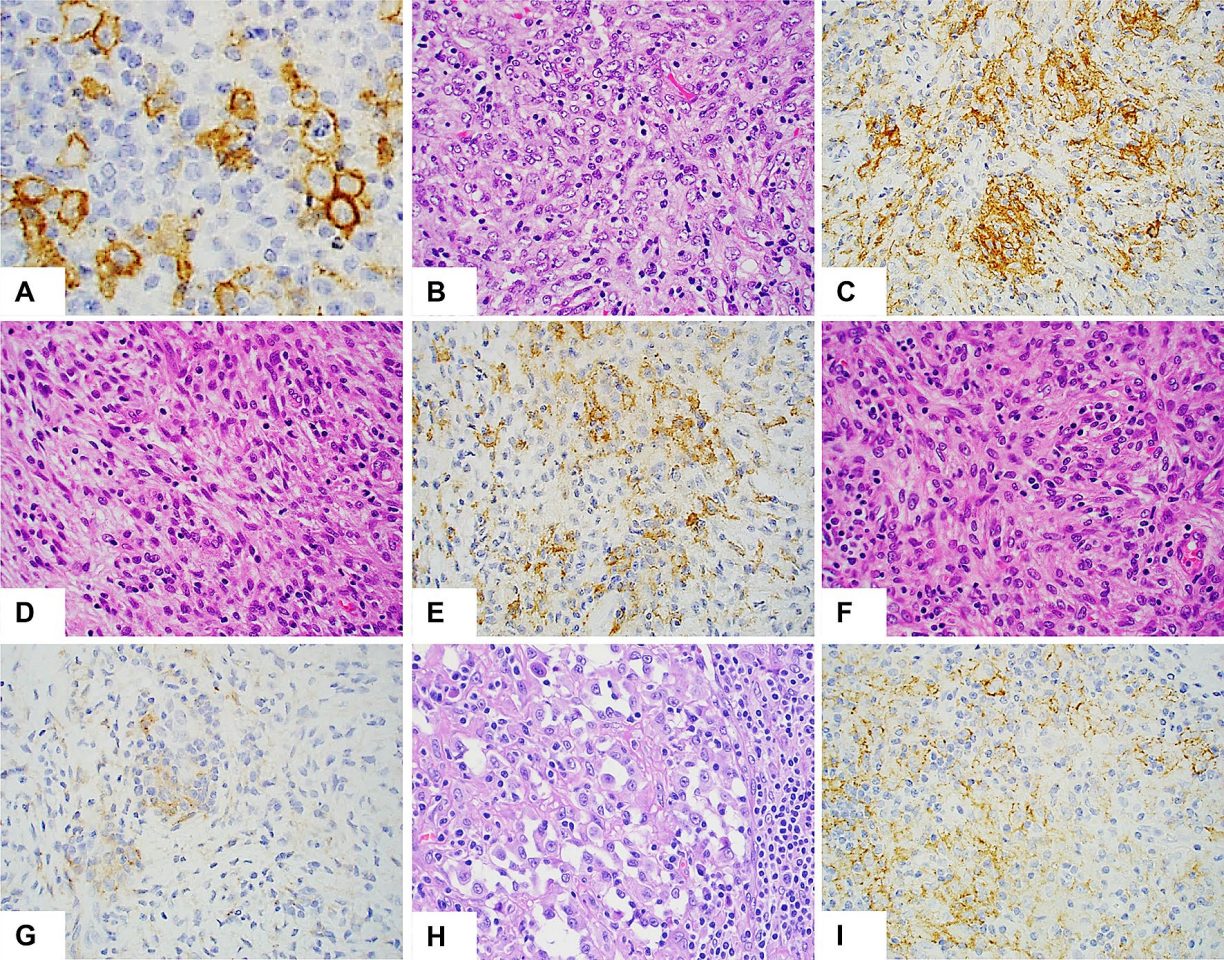
Representative examples of H&E and PD-L1 staining. (**A**) High magnification shows positive membranous staining predominantly on tumor cells (large cells with abundant cytoplasm) along with rare positive immune cells. H&E and PD-L1 staining in case #27 (**B**,**C**, 3 + expression), #11 (**D**,**E**, 2 + expression) and #7 (**F**,**G**, 1 + expression), respectively. (**H**) H&E from case #25 shows that the tumor is composed of clusters of loosely cohesive epithelioid cells exhibiting prominent pericellular clearing surrounded by abundant lymphoplasmacytic infiltrates. (**I**) The tumor cells from case #25 show diffuse PD-L1 expression (3 +) along with 1 + immune cell staining. Original magnification: ×1000 for (**A**); ×400 for (**B**–**I**).

PD-L1 was expressed in a membranous pattern (Fig. 1. A). 58% (21/36) of AFHs showed PD-L1( +) tumor cells, including 4 cases with 3 + expression (Fig. 1B,C), 2 cases with 2 + expression (Fig. 1D, E), and 15 cases with 1 + expression (Fig. 1F,G). 53% (19/36) of AFHs also had PD-L1( +) immune cells, all with 1 + expression (Table 1). 86% (18/21) of AFHs with PD-L1( +) tumor cells also showed PD-L1( +) immune cells, consistent with an adaptive pattern. One case (patient #14) showed immune cell expression of PD-L1, but no tumor cell expression. Overall, PD-L1 expression was seen in 61% (22/36) of AFHs.

### Association between PD-L1 expression and CD8+ T-cell infiltration

**Figure 2:**
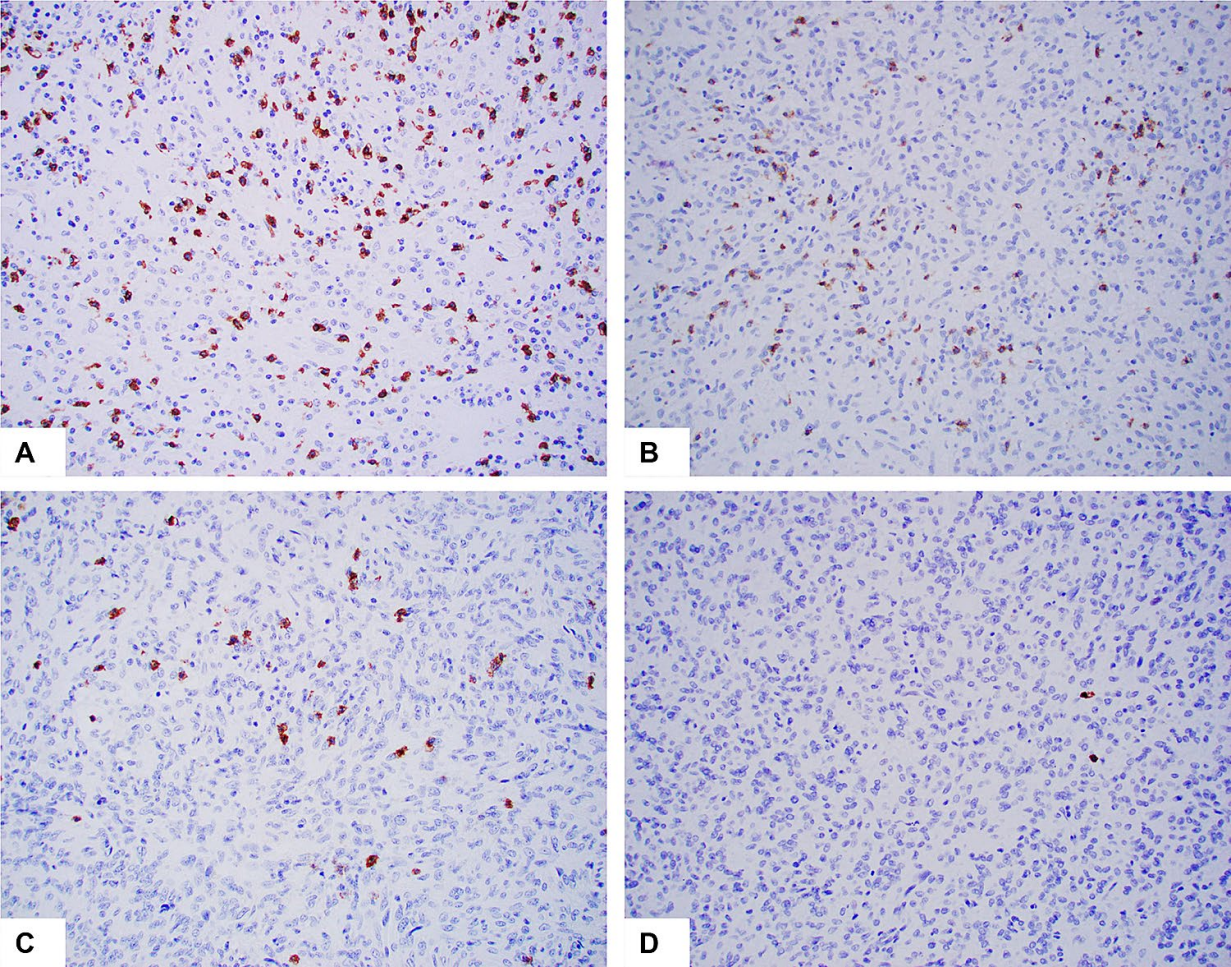
Representative examples of CD8 immunohistochemistry stain. The positive cells show membranous and cytoplasmic staining (brown). There is variable staining in different tumors, ranging from 3 + (**A**, many positive cells) to 0 (**D**, two rare positive cells). (**B**,**C**) 2 + and 1 + staining, respectively. Original magnification: ×200 for all.

Positive CD8 immunostaining highlights cytotoxic/suppressor T cells, which are mediators of adaptive immunity. We found that 81.8% (18/22) of PD-L1 positive tumors had positive CD8 immunostaining (> 10 positive cells per 200 × field). In contrast, only 7.1% (1/14) of PD-L1 negative tumors demonstrated positive CD8 immunostaining. Four PD-L positive tumors had high CD8+ T-cell infiltration (> 100 positive cells per 200 × field) (Table 1). Representative immunostaining of CD8 was shown in Fig. 2. A moderate to strong positive correlation was found between tumor cell PD-L1 expression and CD8+ T-cell infiltration (r = 0.727, *p* = 0.000). The correlation between immune cell PD-L1 expression and CD8+ T-cell infiltration was also significant (r = 0.486, *p* = 0.003). The findings further supported that PD-L1 was expressed in AFH in an adaptive pattern.

### Association between PD-L1 expression and clinicopathologic features

The relationship between the PD-L1 expression and clinicopathological parameters of AFH is summarized in Table 2. There were no statistically significant differences between the clinicopathological features assessed and the status of tumor or immune cell PD-L1 expression. However, there appeared to be a weak trend toward correlation. Both patients with metastasis showed positive PD-L1 expression in both tumor and immune cells. Most notably, the adrenal AFH, which had diffuse metastasis and an *EWSR1-ATF1* fusion, showed epithelioid histology with strong and diffuse PD-L1 expression. (Fig. 1H,I). Three of four patients with paraneoplastic syndrome had positive immune cell expression of PD-L1.

**Table 2:** The relationship between the status of PD-L1 expression and clinicopathologic parameters of AFH.

	Tumor cells			Immune cells		
	PD-L1 ( +) N = 21	PD-L1 (-) N = 15	*p* value	PD-L1 ( +) N = 19	PD-L1 (-) N = 15	*p* value
**Gender**			0.864			0.995
Male	9	6		8	7	
Female	12	9		11	10	
**Age**			0.31			0.738
≤ 8 years	12	6		10	8	
> 8 years	9	9		9	9	
**Tumor site**						0.097
UE	5	6	0.355	5	6	
HN	4	5		2	7	
LE	6	1		6	1	
T	5	3		5	3	
Adrenal	1	0		1	0	
**Tumor size**			0.463			0.985
≤ 2 cm	11	6		9	8	
> 2 cm	10	9		10	9	
**Paraneoplastic**			0.163			0.319
Yes	2	2		3	1	
No	16	7		13	10	
**Metastasis**			0.434			0.323
Yes	2	0		2	0	
No	17	14		15	16	
**Recurrence**			0.126			0.615
Yes	3	0		2	1	
No	18	15	0.864	17	16	0.995

UE, upper extremity; HN, head and neck; LE, lower extremity; T, trunk, *p* value < 0.05 was considered significant.

## Discussion

To our knowledge, this is the first study of PD-L1 expression in AFH by IHC. We found that PD-L1 was expressed in more than half of AFHs with a predominantly adaptive pattern.

The majority of AFHs are curable by complete surgical resection. However, in rare instances, tumors may present with unresectable disease and or develop metastasis. Due to the rarity of unresectable and metastatic disease, there is a limited number of case reports available on the treatment of advanced disease. In a case with unresectable and metastatic AFH, chemotherapy with vincristine, doxorubicin, dactinomycin, and cyclophosphamide achieved a complete response^16^. Six cycles of ifosfamide and doxorubicin followed by three courses of ifosfamide monotherapy were tried with a good response in a AFH with local recurrence and metastases^17^. More recently, Potter et al.^23^ described a clinical response to tocilizumab, an IL-6 receptor inhibitor antibody, in a child with metastatic AFH, whose disease progressed during treatment with traditional chemotherapeutic agents (vincristine, dactinomycin, and cyclophosphamide). In this cohort, all patients underwent primary surgical resection, and three patients recurred locally at follow-up. Two patients had metastatic disease, one of them was an unresectable adrenal gland AFH with diffuse lymph node metastases. Overall, three patients went on to receive systemic chemotherapy. At last follow-up, all patients were alive and two had residual disease.

It is known that AFH is associated with three characteristic gene fusions–*EWSR1-CREB1*, *EWSR1-ATF1*, and rarely *FUS-ATF1*. In this cohort, *EWSR1* rearrangement was documented in 16 of 17 cases analyzed and there were more cases with *EWSR1-CREB1* fusion than with *EWSR1-ATF1* fusion. Unfortunately, so far, no therapies targeting either *EWSR1* or *FUS* fusion proteins are available. Therefore, other novel treatment options for unresectable and metastatic tumors are needed.

PD-L1 is an immune modulator whose interaction with the receptor PD-1 on primed cytotoxic T cells inhibits T cell-mediated tissue damage^24–26^. A number of tumor types utilize this regulatory mechanism to evade immune surveillance by overexpressing PD-L1^26^. Previous studies reported that PD-L1 expression level predicts which patients are more likely to respond to anti–PD‐1 or anti–PD-L1 therapy^27,28^. In this study, the correlation between positive PD-L1 expression, by either tumor cells or infiltrating immune cells, and patient’s gender, age, tumor location, tumor size, or status of metastasis did not reach statistical significance. It might be due to relatively small number of samples in each groups as there is a weak trend toward correlation seen in Table 2. Notably, both instances of metastatic disease in our series showed positive PD-L1 expression in both tumor and immune cells, one of them with strong and diffuse tumor cell PD-L1expression and high CD8+ T-cell infiltration. Additionally, three of four patients with paraneoplastic syndrome had positive PD-L1 expression. The clinical significance of PD-L1 expression in AFH awaits further research, but theoretically, PD-L1 expression by any cell type exerts local immunosuppressive effects leading to evasion of immune surveillance. Therefore, we speculate that immunotherapy with a PD-L1 inhibitor may be a potential option for a subset of AFH patients.

Several anti–PD-L1 antibodies are currently utilized for tumor staining. In this study, we used anti-PD-L1 antibody clone 22C3, which is an FDA approved companion diagnostic antibody for pembrolizumab and a complementary diagnostic antibody for other PD-1/PD-L1 inhibitors^29^. However, our immunostaining was performed by use of Leica bond Autostainer instead of pharmDx assay. Cutoff values (percentage of positive cells) were established on clinical trials for various different carcinomas, ranging from 1 to 50%^29,30^. In this study, the majority of AFHs showed positive staining in > 1% but < 10% of tumor cells (15 cases). Therefore, we arbitrarily used 1% as the initial cutoff. Additional work will be necessary to validate this cutoff value for AFH.

In conclusion, PD-L1 expression was identified in 61% of pediatric AFH cases with a predominantly adaptive pattern of expression. Our findings provide a rationale for future studies evaluating the potential of checkpoint immunotherapy for patients with unresectable and/or metastatic tumor.

## Materials and methods

### Clinical data

Thirty six well-characterized AFH cases from 36 patients with available formalin-fixed paraffin embedded tissues were retrospectively collected from the following institutions: Children’s Hospital Los Angeles, Children’s Healthcare of Atlanta, Washington University in Saint Louis, University of Oklahoma Health Sciences Center, Cincinnati Children’s Hospital and Rhode Island Hospital. The diagnosis of all cases was confirmed by two pathologists (JB and SZ) based on the morphologic features reviewed, and the immunohistochemical and molecular profiles, where applicable, from the submitting institutions.

Ewing sarcoma breakpoint region 1 (EWSR1) gene rearrangements were assessed by fluorescence in-situ hybridization (FISH) using an *EWSR1* break-apart probe in 11 cases, and an *EWSR1* and *CREB1* dual fusion probe in 2 cases. Four cases were analyzed by the use of the OncoKids^SM^ cancer panel^31^. Multiple immunohistochemical stainings such as CD68, CD99 and desmin were performed in most of cases.

### PD-L1 and CD8 immunohistochemical staining and scoring

Representative sections from resection specimen of each primary tumor were stained for both PD-L1 and CD8 immunohistochemically along with appropriate controls by use of a Leica Bond Max Instrument (Leica, Buffalo Grove, IL). Tissue sections (4 μm) were deparaffinized and rehydrated using the Leica Bond Max De-Wax solution. For PD-L1 staining, antigen retrieval was performed with Leica Bond ER2 solution (pH 9.0, EDTA based buffer) for 30 min, then the slides were incubated with anti–PD-L1 antibody (Dako, clone 22C3, dilution 1:50) at ambient temperature for 60 min. Next the antibody was detected using the BOND Polymer Refine Detection kit (Leica), which contains a peroxide block, post primary, polymer reagent, DAB chromogen and hematoxylin counterstain. For CD8 staining, antigen retrieval was performed with Leica Bond ER1 solution (pH 6.0, citrate based buffer) for 20 min, then the slides were incubated with anti–CD8 antibody (Leica, ready-to-use) at ambient temperature for 15 min. Next the antibody was detected using the BOND Polymer Refine Detection kit (Leica), which contains a peroxide block, post primary, polymer reagent, DAB chromogen and hematoxylin counterstain.

PD-L1 was scored based on a semi-quantitative “eyeballing” evaluation by two pathologists (JB and SZ), and cases with discordance were adjudicated by consensus. In this study, PD-L1 positivity was defined as membranous expression in ≥ 1% of either tumor and or immune cells. Tumor cells were distinguished from immune cells on IHC by histologic pattern and size. Tumor cells were typically arranged in syncytial clusters and sheets, and PD-L1( +) tumor cells were much larger and had more abundant cytoplasm than PD-L1 ( +) immune cells. To get a better idea of PD-L1 expression pattern, the percentage of tumor and intratumoral immune cells were evaluated separately, and further scored as 1 + (1% to 10%), 2 + (11% to 30%) and 3 + (> 30%) based on percentages of positive cells. Adaptive PD-L1 expression pattern was defined as PD-L1 positivity seen in both tumor and infiltrating immune cells^32^.

CD8+ T cells in tumor parenchyma (tumor center) were counted manually. Three representative CD8 staining images under 200 × magnification from each tumor were taken. Positive stained cells were counted and averaged. The staining was then scored as 0 (< 10 positive cells per 200 × field), 1 + (10–49 positive cells per 200 × field), 2 + (50–99 positive cells per 200 × field) and 3 + (> 100 positive cells per 200 × field).

### Statistical analysis

The categorical parameters between the PD-L1( +) and PD-L1(−) groups were compared with the chi-square test or the Fisher exact test, where appropriate. The association between PD-L1 expression and CD8+ T-cell infiltration was evaluated using Spearman’s correlation analysis. Statistical analysis was performed using IBM SPSS statistics software, version 17 (IBM Corp, Armonk, NY, USA). All tests were two-sided and *p* values < 0.05 were considered significant.

### Ethics approval

This study was undertaken at Children’s Hospital Los Angeles (CHLA) with the approval of the Institutional Review Board (IRB) (CHLA-18-00278), and independently approved or exempted by the IRB of the participating institutions. All methods were carried out in accordance with relevant guidelines and regulations. This study was granted a waiver of informed consent/assent/permission and a waiver of HIPAA authorization per the Privacy Rule from the parent and/or legal guardian of all patients for publication of identifying information in an online open-access publication by the CHLA IRB.

## References

[1] Costa MJ, Weiss SW (1990). Angiomatoid malignant fibrous histiocytoma. A follow-up study of 108 cases with evaluation of possible histologic predictors of outcome. Am. J. Surg. Pathol..

[2] Fanburg-Smith JC, Miettinen M (1999). Angiomatoid, "malignant" fibrous histiocytoma: a clinicopathologic study of 158 cases and further exploration of the myoid phenotype. Hum. Pathol..

[3] Asakura S, Tezuka N, Inoue S, Kihara N, Fujino S (2001). Angiomatoid fibrous histiocytoma in mediastinum. Ann. Thorac. Surg..

[4] Chen G (2011). Angiomatoid fibrous histiocytoma: unusual sites and unusual morphology. Mod. Pathol..

[5] Khan IS (2019). Primary adrenal angiomatoid fibrous histiocytoma with novel EWSR1-ATF1 gene fusion exon–exon breakpoint. Pediatr. Dev. Pathol..

[6] Konstantinidis A (2019). Intracranial angiomatoid fibrous histiocytoma with EWSR1-CREB family fusions: a report of 2 pediatric cases. World Neurosurg..

[7] Li Q (2014). Primary angiomatoid fibrous histiocytoma in retroperitoneum: report of a case. Zhonghua Bing Li Xue Za Zhi.

[8] Ochalski PG (2010). Intracranial angiomatoid fibrous histiocytoma presenting as recurrent multifocal intraparenchymal hemorrhage. J. Neurosurg..

[9] Ren L, Guo SP, Zhou XG, Chan JK (2009). Angiomatoid fibrous histiocytoma: first report of primary pulmonary origin. Am. J. Surg. Pathol..

[10] Antonescu C (2007). EWS-CREB1 is the predominant gene fusion in so-called angiomatoid fibrous histiocytoma (AFH). Mod. Pathol..

[11] Thway K (2015). Angiomatoid fibrous histiocytoma: comparison of fluorescence in situ hybridization and reverse transcription polymerase chain reaction as adjunct diagnostic modalities. Ann. Diagn. Pathol..

[12] Enzinger FM (1979). Angiomatoid malignant fibrous histiocytoma: a distinct fibrohistiocytic tumor of children and young adults simulating a vascular neoplasm. Cancer.

[13] Matsumura T (2010). Angiomatoid fibrous histiocytoma including cases with pleomorphic features analysed by fluorescence in situ hybridisation. J. Clin. Pathol..

[14] Saito K (2017). Angiomatoid fibrous histiocytoma: a series of seven cases including genetically confirmed aggressive cases and a literature review. BMC Musculoskelet. Disord..

[15] Thway K, Fisher C (2015). Angiomatoid fibrous histiocytoma: the current status of pathology and genetics. Arch. Pathol. Lab. Med..

[16] Bernini JC, Fort DW, Pritchard M, Rogers BB, Winick NJ (1994). Adjuvant chemotherapy for treatment of unresectable and metastatic angiomatoid malignant fibrous histiocytoma. Cancer.

[17] Ogden S (2017). Angiomatoid fibrous histiocytoma: a case of local recurrence and metastases to loco-regional lymph nodes that responded to chemotherapy. Pediatr. Blood Cancer.

[18] Thway K (2008). Angiomatoid fibrous histiocytoma—a review with recent genetic findings. Arch. Pathol. Lab. Med..

[19] Dong H (2002). Tumor-associated B7–H1 promotes T-cell apoptosis: a potential mechanism of immune evasion. Nat. Med..

[20] Taube JM (2012). Colocalization of inflammatory response with B7–h1 expression in human melanocytic lesions supports an adaptive resistance mechanism of immune escape. Sci. Transl. Med..

[21] Topalian SL (2012). Safety, activity, and immune correlates of anti-PD-1 antibody in cancer. N. Engl. J. Med..

[22] Buttner R (2017). Programmed death-ligand 1 immunohistochemistry testing: a review of analytical assays and clinical implementation in non-small-cell lung cancer. J. Clin. Oncol..

[23] Potter SL, Quintanilla NM, Johnston DK, Naik-Mathuria B, Venkatramani R (2018). Therapeutic response of metastatic angiomatoid fibrous histiocytoma carrying EWSR1-CREB1 fusion to the interleukin-6 receptor antibody tocilizumab. Pediatr. Blood Cancer.

[24] Gatalica Z (2014). Programmed cell death 1 (PD-1) and its ligand (PD-L1) in common cancers and their correlation with molecular cancer type. Cancer Epidemiol. Biomarkers Prev..

[25] Patel SP, Kurzrock R (2015). PD-L1 Expression as a predictive biomarker in cancer immunotherapy. Mol. Cancer Ther..

[26] Tumeh PC (2014). PD-1 blockade induces responses by inhibiting adaptive immune resistance. Nature.

[27] Camidge DR, Doebele RC, Kerr KM (2019). Comparing and contrasting predictive biomarkers for immunotherapy and targeted therapy of NSCLC. Nat. Rev. Clin. Oncol..

[28] Kerr KM, Hirsch FR (2016). Programmed death ligand-1 immunohistochemistry: friend or foe?. Arch. Pathol. Lab. Med..

[29] Roach C (2016). Development of a companion diagnostic PD-L1 immunohistochemistry assay for pembrolizumab therapy in non-small-cell lung cancer. Appl. Immunohistochem. Mol. Morphol..

[30] Diggs LP, Hsueh EC (2017). Utility of PD-L1 immunohistochemistry assays for predicting PD-1/PD-L1 inhibitor response. Biomark. Res..

[31] Hiemenz MC (2018). OncoKids: a comprehensive next-generation sequencing panel for pediatric malignancies. J. Mol. Diagn..

[32] Cottrell TR (2018). PD-L1 expression in inflammatory myofibroblastic tumors. Mod. Pathol..

